# Application of Polymer Lubricants in Triboelectric Energy Harvesting: A Review

**DOI:** 10.3390/mi16111195

**Published:** 2025-10-22

**Authors:** Ali Nawaz, Hong-Joon Yoon

**Affiliations:** 1 Department of Semiconductor Engineering, Gachon University, Seongnam 13120, Republic of Korea; 2 Department of Electronic Engineering, Gachon University, Seongnam 13120, Republic of Korea

**Keywords:** lubricant, polymer-lubricant, energy harvester, nanogenerator, triboelectric nanogenerator

## Abstract

The range of lubricant applications has broadened to include multiple sectors, aiming to optimize the operational efficiency of mechanical systems. Given their adaptable friction-reducing properties, lubricants have recently been incorporated into energy harvesting technologies such as triboelectric nanogenerators (TENGs). In such devices, lubricants are essential for mitigating wear, facilitating heat dissipation, eliminating contaminants, and prolonging the service life of mechanically actuated energy harvesters. Notably, emerging developments in sliding and rotational-mode TENGs leverage lubricants to improve electrical output while reducing interface degradation. However, despite significant potential, TENGs still face inherent challenges, including interface friction and energy losses from air breakdown. Recent research indicates that these drawbacks can be effectively addressed by the intentional use of polymer-based lubricants, which contribute to maintaining micro/nanostructured surfaces and minimizing air breakdown, thereby enhancing charge storage capability and increasing device robustness. This review systematically examines the categories, physicochemical attributes, and operational roles of polymeric lubricants used in TENG technology. It underscores their combined function is both primary and support materials to augment triboelectric efficiency. In addition, the article assesses how different lubricants impact device performance and durability, providing a critical analysis of their suitability based on the operational benchmarks of lubricant-embedded TENG configurations.

## 1. Introduction

The performance of triboelectric nanogenerators (TENGs) is strongly affected by their operational environment, especially when subjected to high-frequency or extended mechanical excitation, as friction-induced wear and air breakdown can considerably decrease both device durability and electrical output efficiency [1,2,3]. To overcome these challenges, incorporating lubricants has emerged as an effective approach for reducing interfacial friction and prolonging the operational lifespan of TENG devices [4,5]. Lubricants applied to the contact interface help decrease wear and inhibit processes that lead to charge dissipation, including surface degradation and dielectric breakdown [6,7,8]. Surface wear modifies the interfacial morphology and forms micro-gaps between triboelectric layers, which impairs contact quality and causes a drop in electrical output [9,10]. Conversely, the introduction of lubricant supports smoother contact-separation actions and maintains stable electrical performance [11,12]. Lubricants may also function as protective layers against environmental stressors such as humidity, dust, and temperature fluctuations [13], while certain stimuli-responsive lubricants further shield the device under dynamic mechanical forces [14]. Multiple lubricant formulations have been explored for application in sliding- and rotation-mode TENGs. In one study, Dongwon et al. utilized liquid lubricants to limit surface wear, suppress electrical discharges, and increase surface charge accumulation on PTFE layers [15]. Substances including paraffin oil, squalene, and silicone oil have been used as both electrical discharge inhibitors and agents to boost device longevity. In addition, lubricants derived from 2D materials and synthetic hydrocarbons, such as polyalphaolefin (PAO-4), have shown notable effectiveness in reducing both friction and material wear [16,17,18]. More recently, polymer-based lubricants have gained prominence owing to their adjustable physicochemical properties, adaptability to environmental changes, and broad structural diversity [19]. These encompass substances such as PTFE [17], polystyrene (PS) [20], poly (alkyl methacrylate)s (PAMAs) [21], polymethacrylate (PMA) [22], 3-nitroaniline functionalized olefin copolymer (FOCP) [23], poly(3-sulfopropyl methacrylate potassium salt-co-styrene) P(SPMA-co-St) [24], ethylene propylene diene monomer (EPDM) [25] and poly (methyl methacrylate)-block-poly (dimethyl siloxane) (PMMA-b-PDMS) [26], polyvinylpyrrolidone (PVP) and polyvinyl alcohol (PVA) [27], polyethylene glycols (PEG) [28], vegetable oils [29] and nanocomposites [30] among others. Such polymeric compounds impact lubrication effectiveness by modulating surface tension [31], wettability [32], thermal conductivity [33], and anti-wear properties [34,35,36,37].

Since its introduction in 2012, the TENG has seen notable progress in device architecture, materials engineering, and functional implementations [38,39,40,41]. It has achieved broad application in energy harvesting, wearable sensors, e-skin systems, and biomedical devices [42,43,44,45,46,47]. Nevertheless, persistent problems such as interfacial wear and air breakdown continue to affect both device efficiency and operational longevity [48,49,50,51]. In addressing these challenges, the research community has introduced solutions such as advanced composite materials [52,53], surface texturing [54,55], modifications to device structure [56], and the use of non-contact or soft-contact operational modes [57,58,59,60]. Although these strategies have resulted in certain improvements, fully integrated solutions are still lacking. Consequently, the incorporation of lubricants has been identified as a promising strategy for mitigating tribological issues and enhancing charge generation and device stability [61,62,63].

This review investigates the role of lubricants in TENGs, with a focus on their functions across distinct operating configurations. The dynamics of charge transfer in the presence of lubricants—categorized as solid, semi-solid, or liquid—are analyzed, including observed variations between semiconducting and dielectric contacts. The influence of nanomaterial-based lubricants and stimuli-responsive formulations on TENG performance is also examined. Additionally, a comparative assessment is undertaken, contrasting lubricant-assisted techniques with other friction-mitigation approaches such as surface coating and heat-absorbing barriers. The review closes by discussing existing limitations and suggesting future pathways for the engineered design of lubricant systems compatible with triboelectric energy harvesting.

## 2. Lubricants in TENG Devices

**Figure 1:**
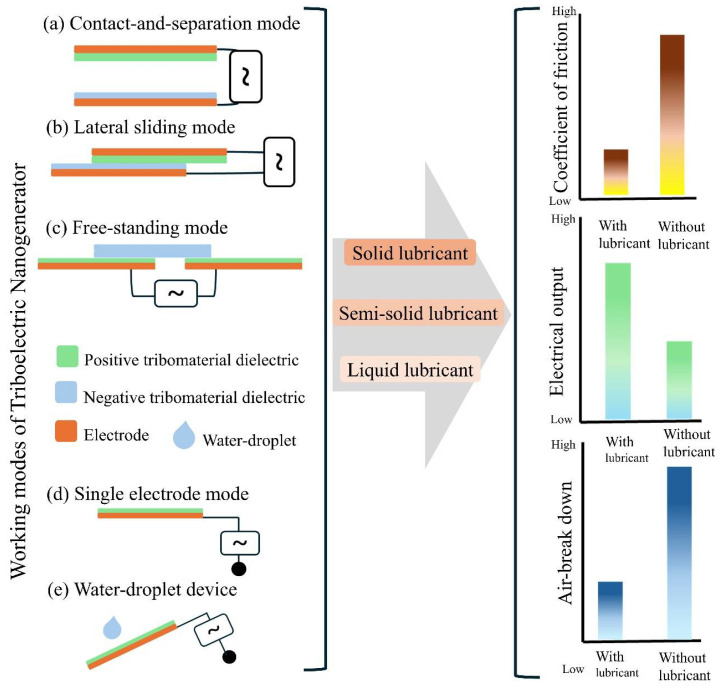
A graphical summary illustrating all operational modes of a TENG device is provided to depict the influence of various lubricant types on friction, electrical performance, and air-breakdown.

Various strategies for interfacial protection have been introduced to optimize the electrical output and performance of triboelectric nanogenerators (TENGs), as schematically illustrated in Figure 1. Detailed analysis reveals that lubricants are effective in preserving the microstructure at the triboelectric interface by preventing air breakdown at the contact boundaries [64]. These lubricants exhibit high performance across all TENG operational modes. Nevertheless, distinct mechanical and tribological properties characterize each mode of operation (such as vertical contact-separation, lateral sliding, single-electrode, and freestanding triboelectric-layer modes), rendering a universal lubricant formulation impractical. The tailored development of various lubricant systems allows for adaptation to the specific operational requirements and interfacial properties of a given TENG configuration. The use of friction-reducing agents has significantly enhanced functional durability in a range of environmental conditions. The following sections present different operational modes of the tested devices.

### 2.1. Contact-and-Separation Working Mode of TENG

**Figure 2:**
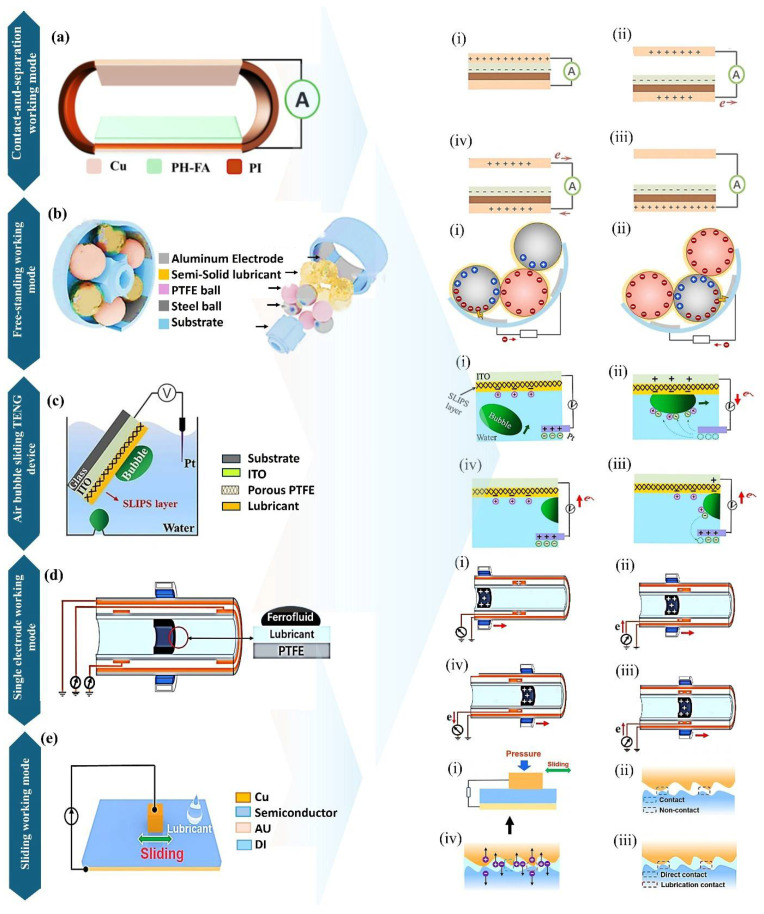
Working modes of TENG devices using various lubricants. (**a**) Contact-and-separation working mode with fatty acids serving as lubricants and (**i**–**iv**) are the charge generation steps of the TENG mode. Reproduced with permission from ref. [63]. Copyright (2023) Elsevier. (**b**) Free-standing working mode of TENG utilizing a semi-solid lubricant is shown graphically and (**i**,**ii**) are the charge generation steps of the TENG mode. Reproduced with permission from ref. [82]. Copyright (2022) Elsevier. (**c**) The air bubble-based energy harvester employs a lubricant surface to facilitate the smooth movement of an air bubble within water and (**i**–**iv**) are the charge generation steps of the TENG mode. Reproduced with permission from ref. [87]. Copyright (2024) Elsevier (**d**) Single electrode working mode of TENG employing a liquid lubricant material and (**i**–**iv**) are the charge generation steps of the TENG mode. Reproduced with permission from ref. [92]. Copyright (2020) Elsevier. (**e**) Sliding working mode of TENG incorporating a liquid lubricant is depicted and (**i**–**iv**) are the charge generation steps of the TENG mode. Reproduced with permission under Creative Commons Attributions (CC BY) license from ref. [16].

This operational mode produces an electric charge through the direct contact of two layers with different tribo-polarities [65,66]. In this setup (Figure 2a), the upper layer is pressed against the lower electrode in the absence of lateral motion, resulting in charge transfer [67]. This working mode serves as the most fundamental approach for examining the charge generation mechanism in TENGs. Commonly, one of the electrodes for charge collection is coated with a dielectric layer and linked to an external circuit [68,69,70]. Through repeated contact and separation of the layers, electricity is generated by the device [71,72]. Continuous contact and separation induce friction and heat, which can adversely affect device performance over time [73,74]. To address interfacial friction, the application of lubricants has been proposed. Despite this, liquid or semi-solid lubricants are generally not suitable for this configuration because their adhesive characteristics hinder proper separation of layers during charge generation. Alternatively, solid lubricants possessing dielectric properties offer improved heat reduction while providing protection to the surfaces, thereby enhancing energy efficiency. In recent investigations, fatty acids have been employed as multifunctional interfacial layers, offering friction reduction while maintaining triboelectric performance (see Figure 2a). As an example, Shen et al. [63] reported a fatty acid-polymer composite TENG utilizing PVDF-HFP that combines low friction with high thermal stability. To further decrease interfacial heating, research has explored the incorporation of thermally conductive additives. For instance, Wang et al. [75] dispersed boron carbide (B_4_C) nanoparticles within a PVDF matrix, achieving optimal electrical output at 20 wt.% loading. This composite not only facilitated superior heat dissipation but also maintained consistent voltage output over 20,000 cycles, signifying improved durability for contact-separation TENGs.

### 2.2. Free-Standing Working Mode

This operational mode utilizes two electrodes and a freely movable upper layer [76,77]. As the upper layer slides over the electrodes, which are connected to an external circuit, electrical charges are generated [78,79]. However, the sliding motion of the free-standing layer introduces substantial friction at the interface, causing more surface damage compared to the contact-and-separation mode [80]. Over time, this friction degrades the TENG’s efficiency by wearing down the contacting surfaces. To address excessive friction, researchers have implemented liquid, semi-solid, and solid lubricants at the device’s charge generation interface [75,81,82]. These lubricants reduce wear, help to fill any voids in the dielectric materials, and displace air entrapped at the surface. Given the continuous sliding in this mode, wear rates are inherently higher than in contact-separation designs. This operational configuration, however, accommodates various types of lubricants—including liquid, semi-solid, and solid forms—to safeguard the sliding surfaces against degradation. For example, in a recent study, a semi-solid lubricant was incorporated into a free-standing mode TENG featuring a ball-bearing mechanism (Figure 2b) [82]. The use of a nonpolar commercial lubricant greatly diminished interfacial friction, permitting stable device performance for 55 h under continuous operation. In another strategy, a self-replenishing film containing squalane liquid lubricant was used to mitigate wear in a similar device. When subjected to mechanical exertion, the lubricant was gradually released from the film, leading to decreased surface wear. Durability testing revealed consistent electrical output from the mechanically adaptive film after 35 days of operation [83].

### 2.3. Water Droplet Energy Harvesting Device

To improve surface mobility, lubricants have been incorporated into water droplet-based triboelectric nanogenerators (TENGs). These lubricants are particularly advantageous for producing hydrophobic surfaces, thereby achieving self-cleaning capability under a range of environmental conditions, such as low temperatures. As a result, lubricants have found widespread use in various water droplet energy harvesting applications [4,84]. At the droplet-surface interface, lubricants play several critical roles—including enabling self-cleaning, facilitating self-healing, shielding against severe environmental exposure, and increasing repellency to water droplets at both high and low temperatures [4,85]. Furthermore, by tuning the surface charge characteristics, lubricants enhance charge generation efficiency from water droplets through improved interfacial conditions. A significant development in the area is the immobilization of liquid lubricants within porous substrates through the slippery liquid-infused porous surface (SLIPS) approach [86].

Researchers developed a porous PTFE (polytetrafluoroethylene) matrix impregnated with liquid lubricant to engineer a high-performance triboelectric interface [85]. This lubricant-infused surface provides twofold advantages, including a marked reduction in friction coefficient at the solid–liquid interface and effective management of dynamic load distribution, which preserves the surface structure under mechanical stress. The method has proven effective in aqueous energy harvesting scenarios, as the lubricated porous interface supports efficient, near-frictionless transport of droplets. In addition, the system preserves robust hydrophobicity, even when subjected to sub-zero temperature conditions [86]. Chen et al. expanded on this strategy by designing a PFPE (perfluoropolyether)-infused PTFE composite tailored for a single-electrode mode TENG device [4]. They impregnated a patterned PTFE substrate with PFPE lubricant, optimizing the surface for seamless movement of water droplets to facilitate electrical generation. The device maintained stable operation at −5 °C. Yan et al. also adopted this method to fabricate an underwater bubble energy harvesting device [87]. Their SLIP-TENG design achieved efficient conversion of kinetic energy from rising air bubbles into electrical output by enabling bubbles to slide across the interface submerged in a water vessel (Figure 2c). Comparative evaluation against a superhydrophobic surface (SHS)-based generator revealed that the SLIP-TBG (slippery lubricant-impregnated porous surface—transistor-inspired bubble energy generator) delivered enhanced performance. It also demonstrated consistent functionality under different pH conditions and varying bubble frequencies. Utilizing lubricants retained in surface pores ensures operational reliability in challenging environments. Despite these advantages, the application of this surface technique in other modes of TENG operation has been limited. Broader integration of this method different across diverse TENG operational configurations may result in the development of more advanced energy-harvesting devices.

### 2.4. Single Electrode Mode

The working mode presents multiple advantages, such as facilitating the efficient fabrication of mechanical energy devices and supporting the development of self-powered sensors capable of tracking objects in motion [88,89,90]. It permits the configuration of TENG devices to generate charges using energy sources characterized by dynamic contact [90]. Charges are produced when an external object encounters, or slides along, the single electrode [91]. To harvest energy from the motion of magnetic materials, a ferrofluid-based liquid was utilized to fabricate a non-contact single-electrode TENG, as illustrated in Figure 2d [92]. Charge generation occurs at the interface between two immiscible liquids within the device. In the structural setup, PFPE (perfluoropolyether) acts not only as the medium for transporting magnetic material but also as a friction-minimizing agent, which promotes unobstructed ferrofluid mobility. The trajectory of the ferrofluid is manipulated by an externally applied magnetic field, thereby enabling free movement of the magnetized liquid inside the hollow PTFE channel. A copper electrode attached to the PTFE surface functions as the collector of the generated charges. The process of charge induction results from the dynamic displacement of the magnetic fluid inside the PTFE tube. In the absence of lubrication, some magnetic material was found to accumulate on the PTFE surface. To address this issue, PFPE oil was added as a lubricant, aiding the effective detachment and unimpeded movement of the ferrofluid. Detailed investigation of the lubricant layer demonstrated that if the film was excessively thick, it hindered the induction of charges, leading to lower output. Despite this, the efficiency of the device may be further improved by examining different lubricant options for use inside the tube.

Additionally, a TiO_2_-doped oleic acid lubricant has been implemented in a single-electrode mode TENG device [93]. This specific liquid lubricant notably reduced interfacial friction, and the device sustained consistent electrical characteristics even after a 3600 s durability evaluation. Evaluation of various TiO_2_ concentrations in oleic acid indicated that 0.1 wt.% delivered optimal lubrication efficiency. In addition to liquid lubricants, solid lubricants have also been integrated into single-electrode TENGs. These solid lubricants exhibit diversity in their compositions, with extensive usage of polymers and composite materials to suppress friction and enhance interface longevity. Typical materials include, PTFE composites [94], silicate compounds [95] and composite-based lubricants [32,33,34]. Furthermore, solid lubricant composite films have been employed at TENG interfaces to achieve a dual benefit: simultaneously reducing friction and enhancing electrical output owing to their synergistic effects. Moreover, silicone oil has served as an interfacial lubricant, attributed to its long Debye length and non-polar fluid characteristics, demonstrating exceptional stability for up to 162,000 operational cycles [6]. A super lubricant, formulated by blending hexadecane with poly-alpha-olefin (PAO) [13], was also investigated. Application of this lubricant to the interface led to marked improvements in device performance. The measurements revealed a significant enhancement, with the open circuit voltage (V_oc_) increasing by 155.6% and the short circuit current (I_sc_) elevated by 140.5%. Simultaneously, the coefficient of friction decreased by 53.0%, clearly indicating that the lubricant not only augmented electrical output but also effectively mitigated mechanical degradation.

### 2.5. Sliding Working Mode

The widely recognized lateral sliding mode, depicted in Figure 2e, operates through the relative motion between two TENG layers that induce triboelectric charge generation [96,97]. Despite its effectiveness, this sliding action introduces friction at the device interface, resulting in heat generation [98] that can degrade overall device performance. To address such issues, multiple lubricant types have been explored specifically for use in this operation mode. Because sliding mode devices are inherently vulnerable to frictional wear and thermal effects, extensive research has centered on the development of lubricant-assisted systems. Notably, rotating TENG configurations have made significant advancements in both friction reduction and enhancement of electrical output.

The adaptability of TENGs operating in sliding mode supports the integration of diverse lubricant types, including solid, semi-solid, and liquid forms directly at the triboelectric interface. Recent innovations have included the application of water@ graphene oxide (GO) lubricants in semiconductor-based triboelectric devices. In tests where the lubricant was introduced between silicon wafers and copper, it preserved both the interface quality and device functionality over 30,000 cycles [99]. In a similar structure, a MXene solution was evaluated to lower the frictional forces at the interfaces. The device structure was made of semiconductor material, and the use of the conductive lubricant improved electrical energy output during the sliding operation of the upper copper electrode [16]. Furthermore, oil and deionized water (DI) were utilized as alternative lubricants, but the MXene solution resulted in a 4.4-fold enhancement in performance. Conductive lubricants, therefore, have proven especially advantageous in semiconductor-based energy harvesting applications. Alongside conventional liquids, a PEDOT:PPS hydrogel film was engineered to act as a lubricating layer. In sliding mode rotation, this organic semiconductor film effectively minimized friction and delivered consistent DC output [100]. The device’s lubrication strategy was not restricted to hydrogels, as several liquid lubricants—including PAO4, squalane, silicone oil, and ethanol—were assessed for their impact on charge output. Among these, ethanol delivered the greatest short-circuit current when assessing electrical characteristics. Additionally, water and alcohol have been utilized as lubricants to further reduce friction in semiconductor-based energy harvesting setups [101]. A variety of TENG device configurations have been designed to convert mechanical energy, with variations in charge generation mechanisms influenced by their operational environment. As previously discussed, the incorporation of MXene as a conductive lubricant significantly benefits semiconductor-based energy harvesting efficiency. Conversely, for polymer dielectric-based TENG devices, conductive lubricants can result in reduced charge generation. This difference emphasizes the necessity of thoroughly understanding the charge transfer mechanism for effective system design.

## 3. Charge Transfer Mechanisms in the Presence of Lubricants and Related Influencing Parameters

### 3.1. Liquid Lubricants-Based TENG Devices

**Figure 3:**
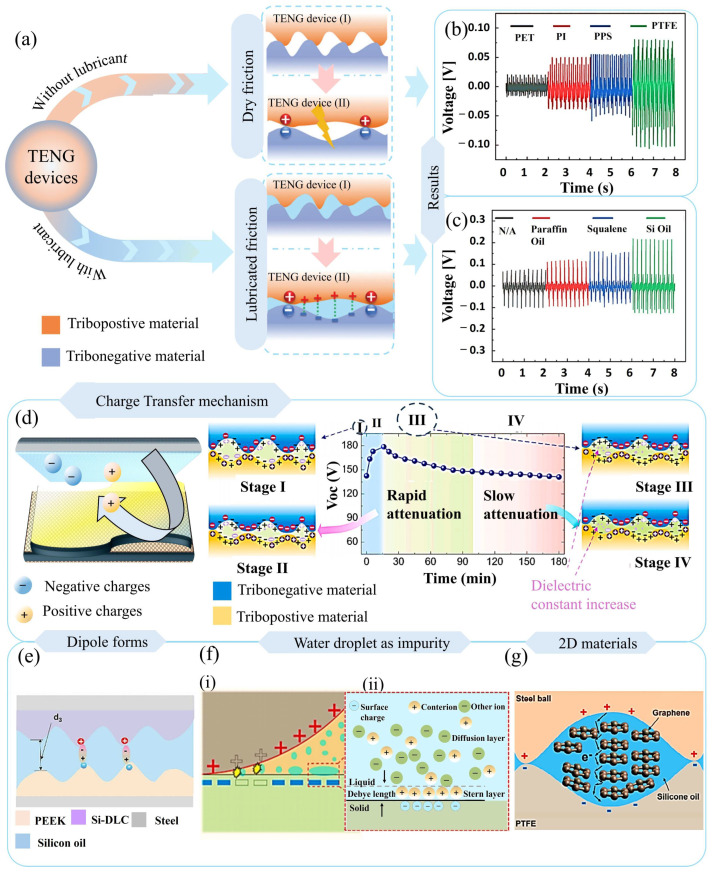
Understanding polarization of lubricants at the interface. (**a**) The operational mechanism of a TENG device with and without lubricant is shown graphically. (**b**) Electrical output is assessed for different polymer surfaces under dry lubricant conditions. (**c**) Upon examining various polymer materials, multiple liquid lubricants were applied to enhance the electrical output. Reproduced with permission from ref. [15]. Copyright (2024) Wiley. (**d**) Various stages of electrical output are depicted graphically. Reproduced with permission from ref. [102]. Copyright (2024) Elsevier. (**e**) Dipole formation at the interface during lubricant application within the device is illustrated. Reproduced with permission from ref. [6]. Copyright (2023) Wiley. (**f**) Presence of water droplets within lubricants induces formation of an electric double layer that (**i**) reduces the surface charges at the interface, and the mechanism is further explained in magnified view (**ii**). Reproduced with permission from ref. [81]. Copyright (2024) Elsevier. (**g**) Experimental results using graphene-doped silicon oil at the interface are shown. Reproduced with permission under Creative Commons Attribution 4.0 (CC BY) from ref. [104].

Due to structural irregularities and surface protrusions on energy harvesting films, the interfaces experience cavitation. These voids initiate air breakdown, generating sparks that dissipate the electrical output of the device, as illustrated in Figure 3a. Conversely, introducing a lubricant can fill these voids and lower both air resistance and charge transfer resistance at the interface, thus facilitating improved charge transfer.

The lubricant additionally forms a protective layer over the surface, safeguarding it from localized external forces. The beneficial impact of lubricants on electrical output has been further corroborated through systematic charge characterization measurements. A TENG device was constructed using the Scott-Russell linkage technique [15], incorporating interdigital electrodes overlaid with a PTFE film to enable sliding-mode functionality. Electrical output measurements taken under dry conditions showed a maximum open-circuit voltage of 0.16 V (see Figure 3b). When lubrication was applied in comparative experiments, the device exhibited enhanced output, highlighting the crucial influence of interfacial states on charge generation. The application of lubricants markedly increases the device’s electrical power, with measured voltages rising to 0.3 V, in contrast to the reduced output observed under dry operation. These experimental findings confirm that interfacial lubrication leads to enhanced electrical characteristics. Comparative testing of various lubricants established that silicone oil outperforms both squalene and kerosene oils (Figure 3c), attributable to its advantageous Debye length characteristics. This improvement is associated with the capability of silicone oil to establish an electrical double layer (EDL) at the interface while avoiding total shielding of triboelectric charges within its Debye length. This property enables effective charge accumulation and retention of device performance. Despite the efficient friction reduction provided by liquid lubricants, their tendency to spill during high-frequency operation constrains their use in sliding-mode TENG systems. This constraint has prompted the adoption of semi-solid lubricants, which address operational stability challenges while still delivering corresponding performance gains.

### 3.2. Grease-Based Lubricants

Liquid lubricants are effective in reducing friction in sliding-mode TENG devices; nevertheless, oil-based lubricants show limited effectiveness due to certain constraints [102]. To overcome these limitations, semi-solid lubricants have been utilized at the energy harvesting interface. Grease, as a representative semi-solid lubricant, plays a crucial role in both friction and heat generation within TENG devices. Furthermore, the influence of grease on electrical performance was investigated by measuring the open-circuit voltage of an energy harvesting device. The semi-solid lubricant demonstrated notable improvement in the electrical output of a TENG device. However, the observed charge generation behavior revealed some notable findings. When grease was applied, the electrical output initially increased but subsequently declined [102]. To explain this decline, the charge generation process was divided into four distinct stages, as illustrated in Figure 3d. During the initial stages (stage I and II), plastic deformation occurs at the interface, resulting in elevated electrical output due to the presence of grease. Further examination of the phases in Figure 3d shows that a rapid decline in voltage output is observed in stage III. During sliding or rotational operation, the grease experiences shear stress at the interface after prolonged use, leading to shear-thinning effects. As the lubricant film becomes thinner, the grease’s dielectric property increases while its viscosity decreases. The improvements in the dielectric properties of the grease contribute to a reduction in voltage output, which is evident in stage III. At stage IV, phase separation of the base oil within the grease occurs. Although this separation gradually increases the dielectric constant of the base oil, it exerts minimal influence on the total electrical output. As a result, stage IV is characterized by only a gradual and modest decline in performance.

### 3.3. Hydrodynamic Lubrication

The charge transfer process in lubricant-based TENG devices is strongly affected by operational parameters, with working load and rotational speed being particularly crucial for device longevity. Hydrodynamic lubrication contributes significantly to distributing the load, as depicted in Figure 3e. When the axial load is kept within a moderate range, a full-film lubrication state is established, maintaining a stable solid–liquid interface [6] that effectively protects the microstructure of energy-harvesting membranes. In this optimal state, the lubricant performs two main functions: it acts as a charge reservoir and simultaneously provides a protective shield for interfacial charges, provided its thickness is constrained within the electrical double layer (EDL) domain. As the applied load increases, the space between layers reduces, promoting more frequent solid–solid contacts and trapping the lubricant within microscopic surface asperities. This results in a mixed lubrication regime that improves electrical performance via two distinct mechanisms. The first mechanism is direct triboelectric interaction at the interface, and the second involves the presence of lubricant-filled cavities that serve as highly efficient paths for charge transfer. Rotational speed introduces additional modulation to these processes, especially under heavy loads [103]. At low rotational speeds, the extent of solid–solid contact is greater. This leads to elevated friction and more pronounced material degradation. By contrast, increasing speed facilitates robust hydrodynamic lubrication effects.

### 3.4. Contaminants in Lubricants

Contamination of lubricants by water droplets and charged particulates has a substantial adverse impact on both the efficiency of charge transfer and the electrical performance of TENG devices. Water ingress not only undermines the lubricant’s physical and chemical stability by accelerating oxidation and decomposition processes but also shortens device lifespan. In addition, conductive particulates interfere with the interfacial charge transfer and the fundamental mechanisms of contact electrification, while both solid and liquid contaminants decrease overall charge transfer capabilities [81]. As demonstrated in Figure 3f(i), the presence of water droplets at the interface accelerates the neutralization of static charges. These droplets generate an electrical double layer (EDL), which hinders the interface’s ability to facilitate effective electrostatic charge generation (refer to the enlarged EDL region in Figure 3f(ii)). Accordingly, lubricant selection for TENG devices must explicitly account for susceptibility to contaminants, especially for applications involving humid or wet conditions where contact with water is unavoidable.

### 3.5. Nanomaterial-Based Lubricants

When carefully chosen impurities that were added to lubricants as additives or nanomaterials can function as agents that enhance performance. Notably, carbon-based additives exhibit outstanding potential, as their excellent wear-resistant characteristics contribute to both improved electrical performance and increased operational lifespan of TENG devices. Recent research indicates that incorporating graphene into silicone oil [104] accomplishes multiple key roles, such as boosting load-carrying capacity, minimizing shear strength, and enhancing charge transfer efficiency. The lubricant containing graphene effectively fills microscopic voids and spaces at the contact interfaces, thereby optimizing the surface interaction and enhancing overall device functionality. Significantly, the 2D graphene structure operates as a highly capable charge transport medium—its specific channeling properties allow more efficient electron movement across the interface (Figure 3g), which leads to marked improvements in electrical output. This versatile strategy illustrates how intentional materials engineering can convert traditional lubricants into functional, performance-boosting constituents for TENG systems.

## 4. Types and Properties of Lubricants

**Figure 4:**
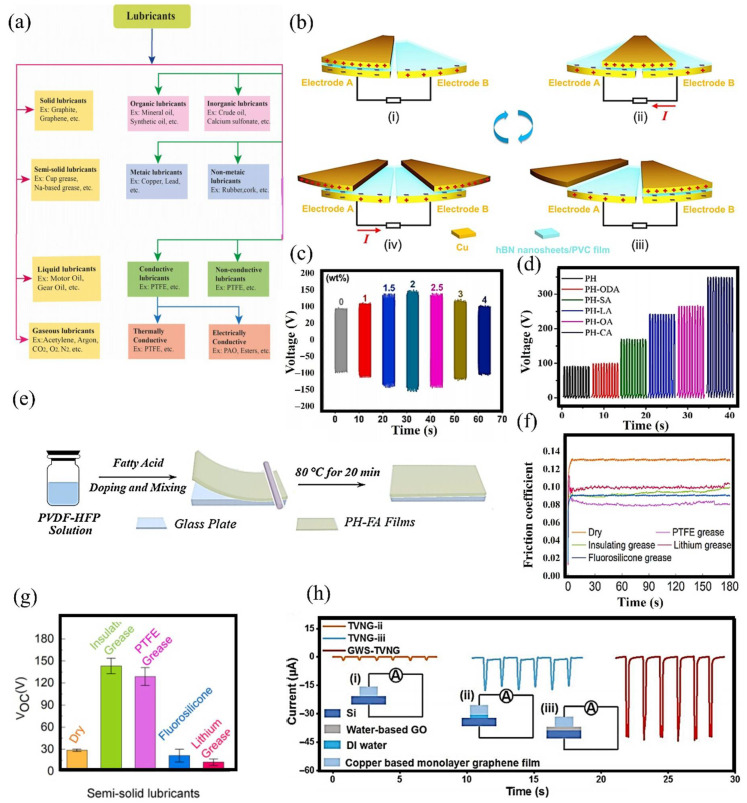
Types of lubricants were utilized in TENG devices. (**a**) Chart illustrating various lubricant types. Reproduced with permission under Creative Commons Attribution 4.0 (CC BY) from ref. [109]. (**b**) A solid lubricant film composed of hexagonal Boron Nitride (hBN) material was employed in a rotating device to harvest energy and the detailed charge generation process was presented in (**i**–**iv**). (**c**) Open-circuit voltage corresponding to different wt.% of hBN films. Reproduced with permission from ref. [53]. Copyright (2024) Elsevier. (**d**) Graphical representation of the electrical output from fatty acids, including octanoic acid (OA), octadecanoic acid (ODA), lauric acid (LA), oleic acid (OA), and capric acid (CA). (**e**) A self-lubricating film was fabricated through doping with fatty acids. Reproduced with permission from ref. [63]. Copyright (2023) Elsevier. (**f**) Coefficient of friction results are presented, including PTFE grease data. (**g**) Voltage output data indicates that PTFE grease generated the highest electrical output. Reproduced with permission from ref. [105]. Copyright (2023) Elsevier (**h**) The output short-circuit current demonstrates that the water-based graphene solution produced the highest electrical output as the output of other devices (**i**–**iii**) shows low output. Reproduced with permission from ref. [99]. Copyright (2022) Wiley.

Lubricants display a wide range of physicochemical properties, which can be systematically classified based on their material state and functional characteristics, as shown in Figure 4a. The initial classification of lubricants is based on their physical state, including solid, semi-solid, and liquid forms. Additionally, they can be further differentiated by their chemical composition into organic, inorganic, metallic, and non-metallic groups. Crucially, lubricants exhibit varying electrical properties that range from conductive to non-conductive. The conductive types can be further classified based on their thermal or electrical conductivity. Selecting lubricants for triboelectric nanogenerator (TENG) applications necessitates a thorough evaluation of their multifunctional roles. Lubricants at the interface can enhance electrical performance through regulated charge transfer processes, increase device longevity by reducing interfacial wear, and diminish frictional heat generation at points of contact. As indicated in Table 1, each lubricant class exhibits distinctive features that influence its appropriateness for TENG applications. The subsequent sections deliver an in-depth evaluation of these lubricant types, focusing on the relationships between their structures and properties as well as their performance indicators in energy harvesting applications. Particular attention is paid to interfacial processes that dictate their efficiency in triboelectric contexts.

### 4.1. Solid Lubricants

Solid lubricants in triboelectric nanogenerators (TENGs) are commonly utilized in the form of composite films, which provide both surface protection and contribute directly to energy generation [75]. Recent research has demonstrated that nanostructured films embedded within polymer matrices, such as polyvinyl chloride (PVC) composites containing hexagonal boron nitride (h-BN) nanosheets [53], offer considerable advancements. These specially designed films display outstanding tribological behavior, resulting in substantial interfacial friction reduction and notable increases in operational lifespan for rotational energy harvesting systems. The structural configuration and operating principle of the device are illustrated in Figure 4b. Based on this configuration, the nanomaterial-based film positioned on the stationary disk not only protects it from frictional wear but also improves the device’s electrical output. The impact of varying h-BN nanosheet wt.% on electrical performance was further analyzed, with open-circuit voltage data for the rotating disk device presented in Figure 4c. Lubricants were either coated onto the charge-producing surfaces or used as additives/fillers within other lubricant forms, such as semi-solid or liquid variants. Furthermore, these coatings functioned as slip layers, which were applied directly to the energy harvesting surface of a TENG device [87]. Sliding layers comprised diverse material blends, where multiple fatty acid types were blended into polymer matrices to create efficient energy harvesting interfaces. For TENG devices relying on contact separation, these composite films proved especially effective, as the fatty acid ingredients improved lubrication at the interface. Experimental results revealed pronounced variation in electrical performance among the fatty acid-based formulations, with the PVDF-HFP/capric acid (PH-CA) composite film demonstrating superior electrical generation, as quantitatively depicted in Figure 4d. The methodology for preparing these polymer-fatty acid membranes is detailed in Figure 4e. This diagram outlines the key steps: blending the polymer matrix with fatty acids, followed by thermal curing at 80 °C for 20 min on glass substrates. Such an optimized fabrication process generated films containing embedded lubricants that substantially lowered interfacial friction while maintaining high triboelectric efficiency.

### 4.2. Semi-Solid Lubricants

Semi-solid lubricants, including nonconductive greases and hydrogels, are frequently used in TENG devices to reduce friction at the contact interface [105,106,107]. Among these options, polymer-based greases such as PTFE grease have been demonstrated to decrease mass loss and interfacial wear, while also enhancing the electrical performance of rotational TENG systems [105]. These lubricants help to limit material degradation and are effective in significantly reducing wear volume [105]. In a similar manner, hydrogels are capable of lowering friction and, at the same time, facilitating electric charge generation at the interface [106]. The performance of different lubricants was assessed by analyzing the coefficient of friction for various greases. PTFE and insulant greases exhibited enhanced friction-reducing characteristics, as shown in Figure 4f. An important function of these lubricants is to provide protection for the energy-generating layers in the device. In addition, their effect on electrical output was evaluated, with Figure 4g indicating that both PTFE and insulant greases yielded notable improvements in the performance of the rotational TENG device. Electrical output measurements obtained using insulant grease verified that the lubricant did not trap charges during device operation. For energy-harvesting devices, it is crucial to sustain high electrical output even with the use of lubricants. Moreover, the reduction in interfacial friction plays a key role in extending the service life of TENG devices.

### 4.3. Liquid Lubricants

Liquid lubricants have demonstrated considerable efficacy in minimizing thermal effects and reducing interfacial wear at triboelectric charge generation surfaces. These lubricating agents include a broad variety of liquid-phase substances, such as alkanes (e.g., hexadecane), aqueous solutions, and diverse oils (PAO-6, rapeseed oil, silicone oil, squalane oil), along with advanced formulations that involve nanoscale and two-dimensional material composites [6,16,100,101,108]. Their utilization is especially beneficial in TENGs operating in lateral sliding or free-standing modes, where the frictional wear rate is significantly higher than in devices functioning in contact-separation mode. Comparative studies indicate that traditional grease-based lubricants often suffer from dielectric instability over prolonged periods of operation, primarily due to the mechanical stress experienced in rotational systems, which ultimately reduces electrical output. Conversely, liquid lubricants exhibit enhanced dielectric stability, making them highly appropriate for sliding-mode TENGs that require reliable long-term electrical output. Moreover, the functional characteristics of these lubricants are amenable to precise tuning through selective modification of additive compositions or the introduction of nanoscale reinforcements. The performance of liquid lubricants can be further optimized by incorporating specific additives or by introducing nano- and 2D materials. For example, as illustrated in Figure 4h, a liquid lubricant comprising water and graphene oxide led to a marked enhancement in the electrical performance of a sliding-mode TENG device.

**Table 1:** Various types of lubricants used in mechanical energy harvesting devices.

Lubricant Type	Working Mode	Isc	Voc	Highlighted Benefits	Major Drawbacks
DI Water	Sliding working mode	0.28 µA	0.4 V	Water functioning as a lubricant significantly enhances electrical output.	The presence of water and ethane leads to a lower electrical output in n-n junction configurations [101].
Lubricant oil (Dupont Krytox)	Single electrode configuration	N.A.	26.4 V	The presence of lubricant provides a slippery surface, facilitating the movement of air bubbles.	The electrical output was lower compared to that of a solid-state energy generator [87].
TiO_2_-doped oleic acid	Single electrode mode	3.8 nA	2 V	The device exhibited improved tribological and triboelectric performance.	An increase in the wt.% of TiO_2_ particles led to greater friction generation [93].
Graphene oxide (GO) in a water-based solution	Sliding working mode	47 µA	0.02 V	The device’s short-circuit performance was enhanced.	The open-circuit voltage remained unchanged at varying sliding speeds [99].
PDMS combined with hollow Si_2_O spheres	Rotational working mode	10 mA/m^2^	260 V	The film demonstrated inherent self-lubricating properties.	The dielectric properties of the composite film were similar to those of pristine PDMS [55].
Silicon oil	Rotational working mode	2.8 nA	0.2 V	Silicon oil, as a non-polar liquid, enhances the electrical output of the device.	A greater length of liquid pockets at the interface leads to reduced electrical output [6].
Mxene Solution	Sliding working mode	53.9 µA	0.227 V	The MXene solution increases the short-circuit current.	The device exhibited a decreased open-circuit voltage [16].
Fatty acid doped polymer film	Contact-and-separation mode	2.81 µA	296 V	The self-lubricating film played a significant role in increasing both the dielectric constant and *β*-phase crystallinity.	Charge dissipation is more pronounced at elevated temperatures [63].
Hexadecane	Rotating working mode	Not available	1380 V	Lubricants suppress air breakdown and enhance the accumulation of surface charges.	Introducing water at a very low concentration (0.01%) into the lubricant leads to a decrease in electrical output [81].
h-BN nanosheets based polymer film	Rotating disk structure mode	142 µA	292 V	The nanosheets’ high thermal conductivity protects tribo-materials and extends the device shelf life in TENG applications.	Adding more than 2 wt.% of nanosheets causes a reduction in electrical output [53].
Grease	Free-standing rotating disk mode	150 µA	179 V	The application of grease eliminates air gaps, which subsequently enhances the device’s electrical output.	Grease with polar properties, such as lithium grease, limits charge generation at the interface [102].
Graphite powder	Contact-and-separation mode	6.0 µA	78 V	The coefficient of friction was reduced as a result of using the graphite material.	The open-circuit voltage exhibits a very gradual increase [110].
Graphene-doped silicon oil	Sliding working mode	9.24 nA	2.23 V	A very low coefficient of friction was attained.	A higher percentage of graphene leads to reduced electrical output [104].

In addition to conventional lubrication methods, various advanced surface engineering techniques have been explored to increase the operational lifespan of triboelectric nanogenerator (TENG) devices. These newer strategies are classified as stimulus-responsive lubrication, which enables dynamic adaptation of interfacial properties in response to varying operational or environmental conditions. Such innovations signify a transition from static lubrication systems to actively responsive interfaces, thereby sustaining optimal tribological and electrical behavior during extended device use.

## 5. Stimulus-Based Surface Lubrication Techniques

### 5.1. Stress-Responsive Technique

**Figure 5:**
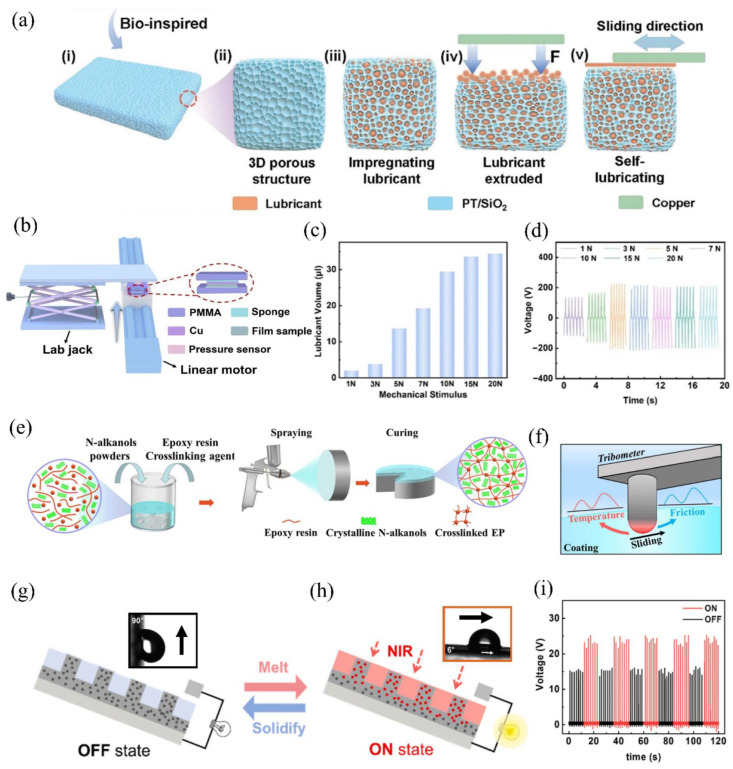
Stimulus-responsive lubricant materials were designed to reduce wear at the interface. (**a**) Graphical depiction of a fabricated porous film and the detailed working mechanism of the as fabricated film is presented in (**i**–**v**). (**b**) Experimental setup for charge generation using the porous film. (**c**) Controlled lubricant release triggered by mechanical stimulation. (**d**) Graph showing the relationship between mechanical force and electrical output. Reproduced with permission under Creative Common Attributes 4.0 (CC BY) from ref. [83]. (**e**) Graphical illustration of a self-lubricating film composed of a smart polymer material. (**f**) Schematic of tribometer devices used to evaluate the properties of smart material-based polymer lubricants. Reproduced with permission from ref. [113]. Copyright (2024) Royal Society of Chemistry. (**g**) NIR photo-responsive mechanism of a TENG device illustrated in both the off state (arrow in the image indicates the inclination of the surface to 90°) and (**h**) ON state (arrows in the image indicate the inclination of the surface to 6°). (**i**) The open-circuit voltage produced by the photo-responsive film confirms the On and Off states of the device. Reproduced with permission from ref. [114]. Copyright (2024) Elsevier.

Stress-responsive lubrication techniques have emerged as highly effective strategies for safeguarding triboelectric interfaces under demanding operational environments. These approaches provide significant advantages, particularly when subjected to excessive top-load conditions, a leading cause of surface deterioration in TENG devices. Noteworthy developments encompass the creation of a porous polymeric film that can both store and release lubricants in accordance with frictional stress. This innovative material features a pressure-sensitive release system, in which cyclic compressive forces activate the expulsion of lubricant from within the porous matrix, resulting in a marked reduction in interfacial friction [83,111]. Importantly, the film retains its self-replenishing properties during idle phases in rotational TENG assemblies, as depicted in Figure 5a. To validate these attributes, a pressure sensor was incorporated to track the applied load, and the occurrence of liquid release was monitored throughout the experiment (Figure 5b). Varying mechanical loads (1–20 N) were imposed, with the film retaining 34 µL of liquid under a 20 N load (Figure 5c). Due to its robust structure and favorable operating characteristics, the film was subsequently integrated into an actual energy harvesting test environment. Across fluctuating loads, the film demonstrated an enhanced electrical output (Figure 5d). Recently, Kaka et al. [112] presented a different strategy utilizing linseed oil-filled microcapsules dispersed within epoxy resin matrices. When subjected to mechanical wear, these microcapsules rupture, enabling the film to dispense oil that decreases interfacial friction. Nevertheless, such lubrication mechanisms have seen limited application within mechanical energy harvesting systems. When designed to correspond with particular TENG configurations and usage scenarios, these lubrication technologies have the potential to substantially increase both the durability and efficiency of such devices.

### 5.2. Thermo-Responsive Lubricants Layers

Recent research by Li et al. introduced a novel self-lubricating composite, where n-alkanols are incorporated within an epoxy resin matrix [113]. This composite employs a distinctive phase-change lubrication mechanism, in which solid n-alkanols convert to a liquid state under frictional stress, thereby effectively lowering both the interfacial temperature and friction coefficient. The epoxy resin matrix fulfills two main roles: it preserves the structural integrity of the n-alkanol constituents and enhances the mechanical robustness of the composite. Detailed material characterization indicated that the solid n-alkanol particles establish crosslinked networks throughout the epoxy resin, and the composite film is produced via spray coating, followed by a thermal curing process (Figure 5e). Tribological testing using a standard tribotester demonstrated that the composite’s properties vary with temperature. Importantly, the composite shows an immediate adaptation to changes in interfacial temperature, where phase transformation of n-alkanols occurs, while the epoxy matrix (with its higher melting temperature) upholds the structure (Figure 5f). The epoxy resin has a higher melting temperature than n-alkanols, providing stability to the matrix even at elevated temperatures. While the composite’s tribological performance has been extensively evaluated, its application in triboelectric systems remains unexplored. Owing to its robust mechanical features, the developed composite film presents good prospects for integration in sliding or rotational-mode triboelectric nanogenerators (TENGs), making it a strong candidate for use in self-powered sensor platforms.

### 5.3. Photo-Responsive Lubricants

Recently, a thermo-responsive lubricant was engineered by integrating paraffin wax with a PDMS and Fe_3_O_4_ composite nanoarray framework, resulting in a light-responsive film [114]. The addition of paraffin wax enabled controlled manipulation of friction at the water droplet sliding interface through the regulation of an external light source. Yun et al. [114] established that this strategy increased the electrical output of a water droplet-driven device by up to 50% when illuminated. In constructing the device, iron nanoparticles were utilized as photothermal agents to enhance heat generation throughout the matrix, whereas PDMS provided the foundational structure. Paraffin wax was subsequently applied onto the micro-structured surface to finalize the device [114]. Ferromagnetic nanoparticles, measuring 8 nm, were dispersed within a micropillar-based composite film. The device functioned based on light-induced temperature modulation at the interface, achieved by exploiting the photothermal properties of semiconductor nanoparticles embedded in the PDMS matrix. Upon illumination, these nanoparticles efficiently converted optical energy into thermal energy, elevating the interface temperature. This temperature increase initiated a solid-to-liquid phase transition in the paraffin wax, reducing surface friction and permitting water droplets to slide with ease. In contrast, without illumination, the paraffin wax reverted to a solid phase, thereby increasing surface adhesion and restricting the movement of droplets. The device demonstrated a rapid and reversible response to light stimuli, with near-infrared (NIR) radiation acting as the external trigger to switch the operational state between ON (melted paraffin, low friction) and OFF (solidified paraffin, high adhesion), as depicted in Figure 5g,h. This technique enabled refined modulation of droplet dynamics, directly impacting the electrical output efficiency of the energy-harvesting system. Voltage differences observed between the ON and OFF conditions, shown in Figure 5i, corroborated the device’s light-responsive electrical behavior.

## 6. Physicochemical Properties of Lubricants

The physicochemical properties of lubricants, such as total acid number, total base number, pour point, viscosity index, kinematic viscosity, oxidation stability, density, and flash point, significantly influence the performance of energy harvesting devices [115,116]. These factors gain particular importance for mechanical power generation systems exposed to harsh environments, as they govern lubricant efficiency, thermal resilience, and operational longevity. The subsequent sections provide an in-depth analysis of these crucial properties.

### 6.1. Total Acidic and Basic Number Characterization

Similarly to other lubricant properties, total acid number (TAN) and total base number (TBN) are essential indicators for assessing lubricant quality. Since lubricants operate over a broad temperature range, they are prone to chemical changes and degradation. Atmospheric oxygen exposure accelerates the oxidation process, resulting in elevated TAN values [117]. This oxidative process diminishes lubricant effectiveness and hastens the deterioration of primary constituents [118]. To address these challenges, lubricants are formulated with performance-enhancing additives—either acidic or basic—to improve stability and operational life. For instance, diesel engine lubricants typically contain basic additives, whereas industrial oils for transformers and turbines often use acidic formulations [119]. Low TAN values generally correlate with increased lubrication efficiency [117], making this parameter vital for selecting lubricants in triboelectric nanogenerator (TENG) applications.

### 6.2. Pour Point

The pour point of a lubricant is the lowest temperature at which it still retains its flow properties before solidification occurs. This characteristic is critical in determining lubricant functionality under freezing conditions, as it reflects the ability of the base oil to remain fluid and deliver continuous lubrication at extremely low temperatures [120]. Although power generation devices are frequently tested under such cryogenic environments, research has shown that their electrical performance often remains stable across wide temperature variations [121]. Choosing lubricants with a low pour point can further enhance system reliability by preventing fluid loss at sub-freezing temperatures, thereby increasing the operational lifespan of energy harvesting devices.

### 6.3. Viscosity and Density

Viscosity is a fundamental property of lubricants that has a pronounced impact on their overall functionality. Ideally, lubricants should display low viscosity at reduced temperatures to ensure smooth engine startup and high viscosity at elevated temperatures to sustain effective load carrying [122]. The viscosity index, representing the rate at which viscosity changes with temperature, is also a crucial parameter for selecting lubricants in devices such as self-powered sensors and energy harvesters. The appropriate choice of viscosity index can prolong device durability and efficiency. Moreover, lubricant density significantly impacts the mechanical behavior of energy harvesting systems. As temperature rises, density decreases, and this variation can influence triboelectric nanogenerator (TENG) performance. It should also be noted that the presence of lubricants adds marginally to the overall weight of an energy harvesting device. Consequently, selecting an appropriate lubricant for mechanical energy conversion devices requires careful evaluation. Additionally, lubricant density directly affects overall system mass, and the use of lighter formulations is generally favored for practical, real-world implementations.

### 6.4. Oxidation Characteristics

Lubricants are essential for reducing oxide formation at interfaces, which remains a significant challenge for mechanical components. Although oxidation poses a substantial issue during operation, the oxidative stability of a lubricant is also crucial in determining the shelf life of mechanical structures. Degradation caused by oxidation leads to increases in the acidity, temperature and viscosity of the lubricant, ultimately affecting its performance [123,124]. Furthermore, acidic oxidation byproducts are capable of reacting with metals, resulting in secondary compounds that further accelerate oxidation processes [125]. To assess oxidation stability, methods such as the Hot Oil Oxidation Test (HOOT) are utilized. In a notable study, to mitigate photo-oxidation effects, the evaluation was performed in a dark oven set at 100 °C for 96 h, with viscosity measured before and after exposure to determine the lubricant’s resistance to oxidation [117].

### 6.5. Flash Point

Evaluating the thermal safety of a lubricant requires determining its flash point, which serves as a fundamental parameter linked to the oil’s evaporation characteristics. The flash point refers to the lowest temperature at which the lubricant emits enough vapor to ignite in the presence of an external flame [117]. Lubricants with higher flash points provide greater thermal stability and enhanced safety, making them preferable for applications involving elevated temperatures. In contrast, lubricants with lower flash points exhibit greater volatility, thereby increasing the likelihood of fire hazards [126]. This characteristic is vital for fire risk evaluation and is especially important in the design of fire-safe energy-harvesting devices.

### 6.6. Tribological Characteristics of a Lubricant

The tribological characteristics of lubricants are fundamental, as they directly impact friction and material wear in triboelectric nanogenerator (TENG) systems. Lubricants that perform well help suppress wear, but extended operational periods may diminish their effectiveness, leading to higher friction levels. Instruments such as the four-ball tribometer are commonly employed to investigate these properties. Moreover, quantitative assessment of specific wear rates is performed using recognized analytical methods [126].

(1) $$K=\frac{wear\;volume\left({mm}^{3}\right)}{sliding\;distance\left(m\right)\times load\left(N\right)}$$

The characterization was conducted by analyzing the scar diameter obtained from four-ball wear test experiments. Furthermore, the coefficient of friction was evaluated using the same experimental approach [127].

## 7. Lubricants Impact on the Different Areas of a TENG Device

Lubricants are fundamental in reducing wear and friction in the moving parts of mechanical energy harvesting systems. Upon application, lubricants occupy voids in the tribo-film, protecting elevated surface features and ensuring stability at energy-conversion interfaces. Moreover, they assist in distributing axial loads and help retain the intricate micro-scale details of tribosurfaces [78]. Beyond their friction-reducing benefits, the conductivity and wettability of lubricants also play a critical role in enhancing the operational durability and optimizing the electrical response of triboelectric nanogenerator (TENG) devices. The following section discusses these mechanisms in greater detail.

### 7.1. Lubricant as Surface Morphology Protector

**Figure 6:**
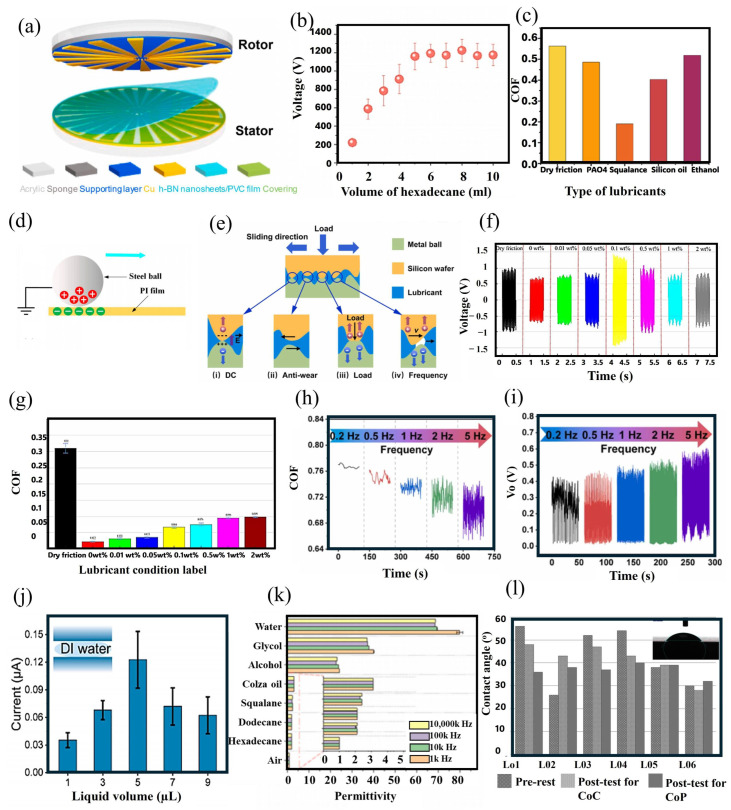
Graphical illustration of friction reduction and electrical output enhancement mechanism. (**a**) Rotating structure apparatus developed to quantify the amount of lubricants. Reproduced with permission from ref. [53]. Copyright (2024) Elsevier. (**b**) The volume of hexadecane affects the electrical output. Reproduced with permission from ref. [81]. Copyright (2024) Elsevier (**c**) Measured coefficient of friction for various lubricants. Reproduced with permission from ref. [100]. Copyright (2023) Elsevier (**d**) Steel-based TENG device configuration. Reproduced with permission under Creative Commons Attribution (CC BY) from ref. [93]. (**e**) Behavior of lubricants at the device interface. Reproduced with permission from ref. [18]. Copyright (2022) Elsevier. (**f**) Electrical output is relative to different lubricant wt.%. (**g**) Coefficient of friction for varying wt.% of lubricants. Reproduced with permission under Creative Commons Attribution (CC BY) from ref. [93]. (**h**) Coefficient of friction measured at different frequencies. (**i**) Electrical output measured across a range of frequencies. Reproduced with permission from ref. [18]. Copyright (2022) Elsevier. (**j**) Effect of water volume as a lubricant on electric charge generation. Reproduced with permission from ref. [101]. Copyright (2024) Elsevier. (**k**) Dielectric properties of various lubricant materials. Reproduced with permission from ref. [53]. Copyright (2024) Elsevier. (**l**) Measured contact angle for each lubricant. Reproduced with permission under Creative Commons Attribution (CC BY) from ref. [127].

Surface modification techniques have demonstrated considerable effectiveness in improving the electrical output of triboelectric nanogenerator (TENG) devices [128]. Micro/nanostructured features, including PDMS pyramids, pillars, interdigital circuits, grating configurations, and circle/x-patterned surfaces, markedly enhance charge generation [53,129,130]. Nevertheless, these structured surfaces are prone to wear during energy harvesting operations. To protect these delicate, charge-promoting structures, various lubricants (such as oils, greases, and solid composite films) are introduced at the contact interface to preserve surface morphology and reduce friction. Notably, some lubricants also actively participate in the charge-generation process. As depicted in Figure 6a, an h-BN composite film coated on an interdigital copper (Cu) electrode fulfills a dual function; it shields the structural integrity of the stationary disk’s electrodes while also enhancing the electrical output in a rotational TENG configuration. When the composite film concentration reaches 2 wt.%, it results in an increased surface charge density compared to pristine PVC, thereby boosting device performance [53]. Another advantage of n-BN nanosheets is improved thermal management, which mitigates heat buildup at the contact interface.

Additionally, the amount of lubricant applied has a pronounced impact on the electrical output of a TENG device. For example, in applications where hexadecane is employed as a lubricant (Figure 6b), optimal electrical output is observed at 8 mL. The principal role of lubricants in TENG devices is to decrease friction. However, their performance effectiveness varies and is influenced by the coefficient of friction (CoF), which differs depending on the lubricant type (Figure 6c). For reliable operation, an appropriately selected CoF is essential to maintain the tribolayer surface structure. Failing to account for this factor can lead to performance degradation rather than performance improvement.

### 7.2. Thin Film Formation of Liquid Lubricants

Liquid lubricants are essential in maintaining the integrity of charge-generating layers within triboelectric nanogenerators (TENGs). These lubricants establish a stable interfacial barrier that reduces frictional wear and hinders damage resulting from direct surface contact. The adsorption behavior of liquid lubricants enables the formation of a consistent thin film at the interface, which is vital for sustaining effective tribological performance [108]. Importantly, adsorption kinetics, such as adsorption rate and capacity, are the primary factors determining the longevity of the frictional interface. The extent of wettability of a lubricant is a significant parameter reflecting its interfacial adhesion and capacity to offer protection. As illustrated in Figure 6d, the adsorbed lubricant film not only preserves interaction between the upper and lower triboelectric surfaces but also imparts mechanical stability under varying loading conditions [131]. In addition, this interfacial film protects micro-structured surface features that contribute to enhanced charge transfer and overall electrical output. However, these micro-structured features frequently experience degradation during high-frequency use or exposure to abrasive contaminants and humidity, resulting in non-reversible performance losses and device failure. High interfacial friction rapidly accelerates the deterioration of these microstructures. Accordingly, the application of protective thin layers is essential to safeguard the interface. The functions of a lubricating layer include maintaining surface morphology and facilitating efficient electron transfer during triboelectric interactions. Notably, the performance of liquid lubricants in dynamic devices depends largely on their rheological properties, which are closely correlated with the system’s operating frequency. Comprehensive interfacial evaluation reveals four principal functions of lubricants in sliding-mode TENGs, as summarized in Figure 6e. These four primary functions are: (i) enhancement of charge generation, as lubricants promote stable electrical output; (ii) reduction in direct surface interactions via boundary lubrication, resulting in the development of a protective film between sliding interfaces; (iii) load distribution, adjusting variable axial loads to prevent localized stress accumulation; and (iv) dynamic regulation of the interface, where lubricant migration at high sliding frequencies introduces small gaps that facilitate further charge generation.

### 7.3. Lubricant to Improve Electrical Output and Co-Efficient of Friction

The chemical composition of lubricants is essential in influencing both the coefficient of friction and electrical output performance in triboelectric nanogenerators (TENGs). In a recent investigation by Shao et al. [93] a single-electrode mode TENG was systematically assessed under diverse lubrication environments using Ti_2_O-oleic acid mixtures. As illustrated in Figure 6f, a detailed analysis of various Ti_2_O weight percentages (wt.%) identified that the optimal electrical output occurred at a 0.1 wt.% concentration. Notably, while the additive improved electrical performance, the associated tribological properties demonstrated an inverse trend. An increase in Ti_2_O content resulted in a higher coefficient of friction, as depicted in Figure 6g. This opposite effect emphasizes the challenges in lubricant formulation, where the friction-reducing capabilities of base oils may be offset by additive-driven changes at the interface. Additional studies explored the dynamic characteristics of lubricated TENG systems over a range of operational frequencies. As indicated in Figure 6h,i, frequency-dependent evaluation showed that lubricants substantially impact both interfacial sliding behavior and electrical generation properties. These observations offer important perspectives on TENG operation under variable conditions. The amount of a lubricant was also shown to affect the device’s electrical output, underlining the necessity of optimizing both the formulation and volume of lubricants for enhanced device efficiency. Recently, Luo et al. [101] introduced a semiconductor-based tribovoltaic nanogenerator where a liquid lubricant was utilized at the friction interface to improve device performance. The experimental analysis included multiple liquid lubricants, such as deionized (DI) water, silicone oil, alcohol, and water-alcohol mixtures. Electrical output measurements determined that the thickness of the lubricant layer played a crucial role, with optimal results achieved at a DI water volume of 5 µL (Figure 6j). Comparative studies revealed that ethanol produced a lower electrical output than DI water. To extend the assessment of lubricant composition, a P-type semiconductor was tested, and the electrical output was measured across various DI water and ethanol blend ratios. It was found that the presence of ethanol reduced the electrical output, indicating a negative impact on tribovoltaic performance.

### 7.4. Conductivity and Dielectric Properties of Lubricants

The electrical conductivity of a lubricant is a key factor in determining the performance of triboelectric nanogenerator (TENG) devices, as it directly impacts their electrical output. In a recent study, Li et al. examined the influence of commercial lubricants on a sliding-mode TENG [132]. Experimental results indicated that highly conductive lubricants, such as castor oil and commercial lubricant oil (CD15W-40), substantially impair TENG performance when compared to low-conductivity alternatives like squalene. This decrease in output results from the neutralization of triboelectric charges at the interface—caused by an excess of charge carriers in conductive lubricants—which restricts effective charge transfer [133,134]. Additional evidence is provided by studies on semi-solid lubricants in rotational TENGs, which showed that insulating grease promotes enhanced electrical output, while conductive types reduce system performance. For instance, in a grease-lubricated triboelectric instantaneous angular speed sensor (GL-TEIASS), dielectric grease delivered greater output owing to its electrically insulating nature [105]. The dielectric properties of lubricants are therefore of great importance, as lubricants with high permittivity can increase charge induction. However, lubricants with high dielectric loss tend to result in degraded performance. It is particularly notable that lubricants with low dielectric constants, such as PTFE grease, may improve device output [105]. Comparative studies of liquid lubricants also verified that hexadecane, which has a low permittivity, outperforms high-permittivity substances like water (Figure 6k). These distinct properties highlight hexadecane as a promising lubricant for TENG applications.

### 7.5. Wettability of Lubricants

The wettability properties of lubricants are determined by their capacity to adhere to angular surfaces. These properties are essential for reducing wear in energy devices. Optimal wettability facilitates prolonged lubricant retention on surfaces. This parameter is especially critical under dynamic operating conditions, where shifts in position or exposure to harsh environments can cause the lubricant to become displaced. Wettability is quantitatively evaluated using contact angle measurements [123]. A lower contact angle (<90°) signifies improved surface affinity (Figure 6l), while hydrophobic surfaces (contact angle > 90°) demonstrate reduced wettability and less effective lubricant retention. The interfacial interactions of lubricants depend on two primary forces: adhesion (liquid-solid interactions) and cohesion (attractive forces within the liquid). Together, these forces dictate lubricant stability on tribolayers, with increased adhesion improving surface coverage and cohesion, maintaining droplet cohesiveness. In TENG device manufacturing, the compatibility between the tribomaterial and the lubricant is essential for enhancing device performance. Inadequate adhesion from material mismatch not only diminishes electrical output but also expedites device degradation. This results in decreased energy harvesting efficiency and shorter operational life. Consequently, a careful selection of tribomaterials based on wettability attributes is key to maximizing both TENG performance and durability.

## 8. Comparison of Lubricants with Other Techniques to Improve the Performance of a TENG Device

### 8.1. Thermally Conductive Materials

**Figure 7:**
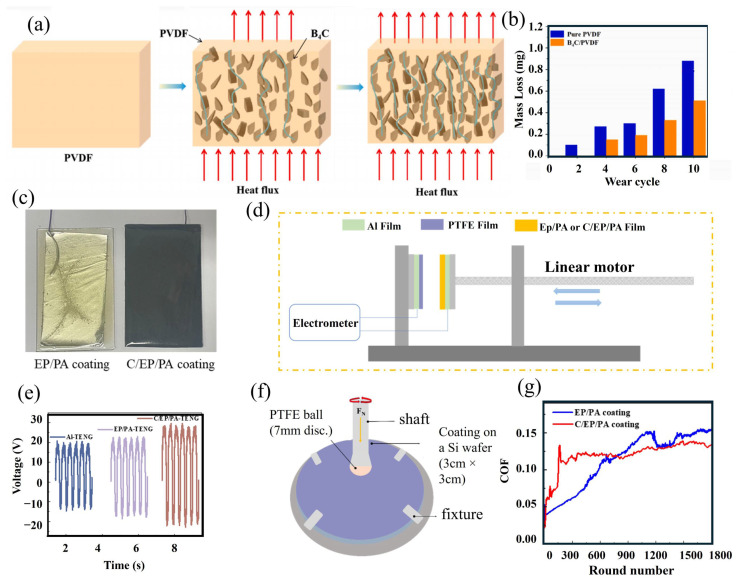
Smart materials were employed in the composite film of a TENG device. (**a**) Diagram showing nanomaterial-based polymer for interfacial heat control. (**b**) Mass loss attributed to friction. Reproduced with permission from ref. [75]. Copyright (2024) Elsevier. (**c**) Polymer film with graphite for wear control. (**d**) Schematic of TENG system utilizing graphite-based materials to generate charge and blue arrows indicate the to-and-fro motion of linear motor. (**e**) Electrical output from the composite film containing graphite. (**f**) Graphical depiction of a wear characterization apparatus. (**g**) Coefficient of friction measured for the graphite-based device. Reproduced with permission from ref. [110]. Copyright (2024) Elsevier.

Heat generation and frictional forces at sliding interfaces present significant obstacles to the durability of energy harvesting devices. To address thermal degradation, thermally conductive particles may be introduced into polymer matrices to regulate interfacial temperature by dispersing excess heat. Wang et al. [75] validated this strategy by integrating boron carbide (B4C) nanoparticles into a polyvinylidene fluoride (PVDF) matrix, which improved the composite material’s thermal management properties. Their investigation systematically assessed the impact of thermal conductivity on interfacial heat dissipation and found that a 20 wt.% B4C-PVDF nanocomposite produced optimal electrical performance (Figure 7a). The presence of thermally conductive fillers enhanced the stability of the device’s voltage output over 20,000 cycles and effectively reduced wear-induced mass loss (Figure 7b), demonstrating the essential function of thermal management in maintaining interfacial stability. In addition to established lubrication methods, these thermally conductive nanocomposites provide a compelling solution for controlling heat buildup at tribological contacts. The combined contributions of increased thermal conductivity and wear resistance extend the service life of energy harvesting layers by lessening both thermal degradation and physical wear. The results emphasize the necessity for innovative material engineering to improve the reliability and performance of triboelectric energy harvesting systems in harsh operating conditions.

### 8.2. Carbon-Based Materials

Graphite has been widely investigated as a friction-reducing additive in polymer composite films due to its advantageous tribological and electrical characteristics. In a recent study, Yang et al. [110] fabricated a water-driven triboelectric nanogenerator (TENG) utilizing graphite as a filler within an epoxy-polyamide matrix to form the energy harvesting layer (Figure 7c). The device employed the graphite-based composite film as a tribo-positive electrode operating in a contact-and-separation configuration (Figure 7d). Performance comparison indicated that the graphite-based TENG achieved substantially improved electrical output compared to the graphite-free variant (Figure 7e). This enhancement stemmed from the superior dielectric properties provided by the carbon filler, enabling consistent electrical generation. For further analysis of tribological attributes, a PTFE ball-based 3D profilometer was utilized (Figure 7f). Moreover, tribological assessment established that the composite without graphite was susceptible to wear, while the graphite-reinforced film offered improved friction-reducing capabilities. At the outset, the CoF was elevated due to surface roughness, yet following 750 cycles, the graphite-composite film recorded a noticeably lower CoF (Figure 7g). These results highlight the composite film’s contributions to extending durability and optimizing energy harvesting performance in TENG applications.

### 8.3. Microhardness Property of a Composite Film

Nanocomposite films display microhardness characteristics that are essential for mitigating wear at the sliding interface in energy harvesting films. The addition of a lubricant material further diminishes surface wear by introducing a protective lubricating layer. Nevertheless, extended operation of triboelectric nanogenerator (TENG) devices can result in lubricant depletion, leading to direct frictional contact between tribolayers. Under such conditions, the microhardness property of the composite film can enhance resistance to wear. This strategy has been applied to a sliding-mode TENG device, yielding impressive outcomes. In particular, a composite film was engineered by integrating BaTiO_3_ (BTO) nanoparticles into a polyimide matrix. The device achieved a 75% decrease in mass loss with castor oil used during the energy harvesting operation. Comparison of surface topography demonstrated that BTO nanocomposite films possess enhanced wear resistance under frictional forces. Microhardness measurements, reported in megapascals (MPa), verified that increasing the nanoparticle weight percentage (wt.%) resulted in improved film microhardness. These results indicate that such nanocomposite films are highly suitable for devices deployed in demanding conditions where lubricant loss cannot be avoided.

## 9. Exiting Gaps and Challenges

There is a notable scarcity of research focused on evaluating the effectiveness of lubricants in mechanical energy harvesting devices. Although lubricants exhibit diverse properties, TENG devices face specific constraints regarding the applicability of various lubricant materials. As previously discussed, the primary purpose of lubricant materials is to minimize heat generation and wear at the interface. In addition, solid, grease, hydrogel, oil, and nanomaterial-based lubricants are known to have properties that can dissipate charges. Nevertheless, an in-depth examination of these lubricants remains limited. The additives contained in lubricants can sometimes have effects that contradict the intended performance outcomes. Additionally, unresolved issues and research gaps are categorized as follows.

### 9.1. Lubricants According to the Working Mode of TENG Device

Selecting suitable lubricants is essential for maximizing the long-term reliability and efficiency of triboelectric nanogenerators (TENGs). Due to the varying operation modes of TENGs—including contact-and-separation, in-plane sliding, free-standing, and single-electrode arrangements—lubricant choices should be compatible with each specific mode. For example, contact-and-separation TENGs are susceptible to performance degradation if liquid or semi-solid lubricants are used, as these can lower electrical output and negatively affect device structure. For these devices, solid lubricants are generally the preferred option. In contrast, sliding and free-standing TENGs, which experience higher frictional forces at the charge generation interface, commonly require liquid or semi-solid lubricants to effectively reduce wear and sustain stable performance. Moreover, the integration of stimulus-responsive lubricants provides a promising approach for dynamically enhancing TENG performance across various working conditions.

### 9.2. Charge Transfer Efficiency of Lubricants

The interfacial lubricant plays a critical role by storing charges and supporting efficient charge transfer. In certain applications, lubricants can also serve as electron donors. However, experimental results indicate that lubricant materials may undergo significant charge depletion when exposed to ambient conditions during a TENG’s energy harvesting process. When lubricants demonstrate inadequate charge retention, this can result in pronounced losses in TENG electrical output. Therefore, thorough examinations of charge transfer mechanisms involving different lubricants are crucial for making informed selections of lubricant materials. The potential of lubricants to effectively preserve and support the transfer of charges is a key consideration in maximizing TENG device output. Despite this importance, a limited research base on lubricant charge transfer efficiency remains a substantial obstacle to the advancement and identification of lubricants that can simultaneously boost TENG output and minimize charge loss.

### 9.3. Lubricant Additives Impact on Electrical Output of a TENG Device

Lubricants comprise various additives tailored to their specific functional roles. Nevertheless, the lubricants used in energy harvesting devices have not been systematically classified according to their additive makeup. Although these additives significantly affect friction and charge generation mechanisms in mechanical energy harvesting systems, the process of additive selection is often neglected. Additive selection needs to be optimized based on the operating principles of TENG devices. Furthermore, insufficient attention to the compatibility between base oil and functional additives leads to inappropriate lubricant choices. Additionally, non-polar, low dielectric constant, and low viscosity keeper additives are often prioritized during lubricant selection.

## 10. Conclusion and Future Perspective

Lubricants have recently attracted considerable research interest due to their multifunctional capacity to protect interfacial surfaces while also enhancing the electrical performance of mechanical energy harvesting devices. As discussed at length, enhanced device output can be traced to several critical lubricant characteristics. These features include the ability to form a thin protective layer at the interface, which reduces frictional losses and limits heat generation effectively. Moreover, lubricants reduce mechanical wear by optimizing axial load management, a critical parameter for device reliability during operation. The physico-chemical properties of lubricants were highly contributed in the smart selection of a friction-reducing agent to protect the operational life of mechanical energy harvester. Although these characteristics of lubricants are often oversighted, that results in poor performance. There are several functions that a lubricant performs at the charge generation interface. The formation of a protective layer in a sliding mode TENG device was the prominent function of a lubricant. The protective function of lubricants is directly linked to their thin-film forming capability. In addition, stimulus lubricant techniques highly contributed to reducing wear at the interface. These techniques can be a future lubricant-based TENG device fabrication technique. Future research aimed at achieving optimal device performance should prioritize the development of advanced composite-based lubricant materials and the incorporation of stimulus-responsive lubricants. These advancements are likely to greatly improve operational lifetime and boost electrical output efficiency.

### 10.1. Physicochemical Properties of Lubricants Utilized in TENG Devices

Lubricant materials are vital in mechanical systems, and their importance extends to energy harvesting applications due to their contribution to the enhanced durability of TENG devices. Despite established benefits for device lifetime, the physicochemical parameters of lubricants have not received sufficient scrutiny in the context of TENG applications. While lubricants are characterized by diverse analytical techniques, essential attributes such as flash point, pour point, and viscosity are frequently disregarded during selection for TENG systems. Although TENG devices often employ low-viscosity lubricants, their propensity to adhere to charge generation surfaces may hinder optimal device function. Therefore, a comprehensive understanding of lubricant physicochemical properties will be vital for informed material selection in future TENG device design.

### 10.2. Instantaneous Energy Conversion Efficiency

Minimizing friction and maximizing the electrical output of TENG devices have been effectively addressed through the application of advanced lubricants. Nonetheless, obtaining quantitative data remains essential for thorough performance evaluation. Moreover, comparing the efficiency of distinct lubricant materials presents a significant challenge when assessing the overall performance of lubricants. This challenge was overcome by utilizing robust quantitative analytical methods. The approach provides an essential metric for quantitatively evaluating lubricant performance [13]. A primary characterization metric is the ratio between electrical output power and frictional input power. Findings from this analysis confirm that lubricant-enhanced TENG devices deliver improved efficiency relative to devices without lubrication. In addition, this quantitative assessment method is recognized as an essential measurement approach for advancing and refining lubricant-based TENG devices.

### 10.3. Structural Modification of Tribolayers

To minimize interfacial friction across the different operating modes of TENG devices, structural modifications play a crucial role in enhancing device longevity. Recently, the development of slippery liquid-infused porous surface TENGs (SLIP-TENGs) has enabled advances in water droplet energy harvesting [86]. The porous membrane design is also a promising choice for application in alternative working modes of TENG devices. Incorporating energy-harvesting membranes into contact-separation and single-electrode-based devices could further reinforce the durability of these systems under various operational conditions. Additionally, the comprehensive exploration of diverse lubricants, particularly stimulus-responsive types, across all TENG working modes is still lacking. Therefore, the adoption of targeted surface modification strategies using lubricant materials in energy-harvesting devices can substantially improve their electrical performance and operational lifespan.

### 10.4. Charge Transfer Mechanism

In-depth investigation of the charge transfer mechanism at interfaces involving lubricant materials is vital. Research has shown that conductive lubricants can suppress charge buildup in polymer dielectric-based TENG devices. However, for semiconductor-based energy harvesting systems, MXene-based conductive lubricants have been observed to increase electrical output. Further, experimentation with both positive and negative semiconductor-based devices indicates that the application of the same conductive lubricant does not result in reduced performance. As such, a comprehensive exploration of the interfacial charge transfer mechanism is essential and will advance our understanding of the charge transport processes in the presence of different lubricants. Ultimately, such analytical models can inform the design of lubricants with opposite polarities, thereby extending the operational lifespan of mechanical energy harvesting devices.

### 10.5. Low Permittivity Materials

In developing lubricant materials for energy harvesting devices, it is crucial to select additives and base materials with low permittivity. Regardless of whether the lubricant is solid, grease-based, hydrogel-based, oil-based, or water-based, achieving optimal performance requires specific operating conditions. Owing to their low permittivity, these materials improve electrical output by decreasing dielectric losses. In contrast, high-permittivity components elevate the lubricant’s dielectric constant, thereby diminishing the device’s overall efficiency. Furthermore, lubricants, additives, and base materials with polar characteristics reduce electrostatic induction at the interface, while non-polar lubricants weaken the interfacial electrostatic field strength, thus limiting electrostatic charge dissipation. Taken together, these results establish essential strategies for designing advanced lubricant materials tailored for TENG devices.
